# Workforce Recruitment Through Pre‐Nursing Vocational and Education Training Schemes. A Qualitative Evaluation Through a Social Capital Lens

**DOI:** 10.1002/nop2.70159

**Published:** 2025-02-09

**Authors:** Marion Waite, Jane Appleton, Kathleen Greenway, Robert Crookston, Sanj Nathoo, Catherine Henshall

**Affiliations:** ^1^ Oxford School of Nursing and Midwifery, Faculty of Health and Life Sciences Oxford Brookes University Oxford UK

**Keywords:** pre‐nursing, social capital, vocational education and training, workforce recruitment

## Abstract

**Aim:**

To evaluate learners' and stakeholders' experiences and perspectives of two models of pre‐nursing vocational education and training (VET) schemes on nursing workforce recruitment.

**Design:**

Qualitative exploratory approach.

**Methods:**

This qualitative study comprised online, semi‐structured interviews of nine stakeholders and eight trainees from one United Kingdom region in which two pre‐nursing VET scheme models were implemented. Interview data was thematically analysed and interpreted through a social capital theory lens.

**Results:**

The interviews uncovered three key themes concerning the participants' experiences and perspectives on workforce recruitment: motivations and expectations, relational aspects of curriculum design and delivery and future possibilities. Established social structures were reported to be connected to education and clinical boundaries, enabling learners to build relationships with staff and patients and influencing their identity and career choices in healthcare. Limited placement opportunities hindered clinical learning, trainees from non‐traditional backgrounds were underrepresented, and the validity of the academic preparation was unclear.

**Conclusion:**

The study makes a novel contribution by explaining how social assets can be created for all parties through two pre‐nursing VET models when stakeholders' expectations and motivations align with learners' aspirations for a vocational route into nursing. This potentially leads to recruitment into nursing and healthcare pathways. The study's insights also showed a lack of recognition of this route to nursing and a lack of standardisation in access and educational delivery. The findings have implications for policy and educational practice. A deeper understanding of the socioeconomic factors affecting learners' nursing and healthcare career choices can enhance existing knowledge. Furthermore, studies are required to compare with other regions nationally and internationally to determine how pre‐nursing VET schemes significantly address the global nurse recruitment crisis while considering local social and economic contexts.

**Reporting Method:**

Consolidated criteria for reporting qualitative research (COREQ).

**Patient or Public Contribution:**

Stakeholder consultation during the study's design phase influenced the development of the research questions. Presenting the findings at a regional stakeholder workshop highlighted the key discussion points reported in the paper.

## 1 Introduction

Globally, creating flexible, accessible pathways into nursing is driven by societal factors, with nurses in short supply across international health and social care settings (RCN 2022). The International Council of Nurses (ICN) estimated a shortage of nurses in 2019–2020, which was further exacerbated by the COVID‐19 pandemic (Buchan et al. 2022). During the pandemic, burgeoning pressures tested nursing workforce morale and increased burnout, leading to a global exodus of nurses.

The ICN recommends increasing the number of new nurses to maintain high health service delivery and patient care standards. However, challenges in recruiting and retaining nursing students exacerbate existing problems in the workforce (Eick et al. 2012). Common reasons for student attrition include psychological and social issues and unpreparedness for the realities of nursing (Bakker et al. 2019). Pre‐nursing vocational education and training (VET) can provide individuals with practical experience and the right qualifications to access formal nurse apprenticeships or pre‐registration nursing programmes (Smith et al. 2015). This early exposure to nursing may reduce the risk of attrition and any related social and psychological problems associated with coping with the role.

Pre‐nursing VET schemes respond to workforce planning needs while aiming to widen participation (Culley and Genders 2003; Kyle et al. 2020; Norman et al. 2008). Stakeholders in education and healthcare organisations aim to design and implement programmes that meet the needs of learners while also enabling workforce capacity building. They recruit, design curriculum, assess learning and oversee clinical placements. The contexts in which stakeholders implement schemes and their success and benefits, such as growing the next generation of staff and opening up nursing careers, require a better understanding. The context of pre‐nursing VET schemes and their intended benefits, such as increasing recruitment to the nursing workforce and widening participation, lacks evidence. In this paper, through a social capital theory lens, we report on a qualitative evaluation of pre‐nursing VET schemes in the UK from stakeholder and learner perspectives on workforce recruitment. This can help aid understanding of stakeholder and learner experiences concerning the factors that influence recruitment to nursing and other healthcare professions.

## 2 Background

In several countries, initial training to enable access to healthcare professional education is an established part of secondary school education. In Switzerland (Grønning and Trede 2019; Trede and Schweri 2014; Zittoun 2003) and Finland (Rintala and Nokelainen 2020), pre‐nursing VET schemes are embedded components of formal upper‐secondary education. The USA implemented pre‐nursing VET schemes in response to the global nursing crisis (Zamora and Martínez Rogers 2020), and in Australia (Fawcett 2010), there is a focus on initial VET in nursing to support entry into nursing programmes for individuals from diverse backgrounds.

Pre‐nursing VET schemes in the United Kingdom (UK), also known as ‘nurse cadet’ schemes, have an inconsistent history and are less embedded than in some countries. They became available for 16‐to‐19‐year‐olds as an alternative to post‐16 education in the 1970s (Culley and Genders 2003; Draper et al. 2004; RCN 2021), offering vocational‐level qualifications (GOV.UK 2022) and a pathway into nursing programmes and apprenticeship schemes. Other entry routes include level three qualifications such as Advanced Levels (A level), Business and Technical Education Council (BTEC), Access courses, T levels and Pre‐nursing VET schemes via an education or workplace route. Following level three study, pre‐registration BSc programmes can be accessed. Nurse associate apprenticeships (a paid programme for work/study) were also introduced in 2017. After completing a Nursing Associate Apprenticeship, a minimum 18‐month adult nursing BSc programme can be undertaken with recognition of prior learning (RPL). The 3‐year BSc adult nursing degree and the RPL programmes lead to registered adult nurse status.

Pre‐nursing VET schemes for 16‐to‐18‐year‐olds in the UK have helped alleviate the response to the nursing workforce recruitment crisis and were rejuvenated in 2019 after a prolonged abeyance. Flexible nursing routes allowed pre‐nursing VET trainees to choose between work, apprenticeship, and tertiary education (Foster 2018; GOV.UK 2022; Orr and Terry 2023). The NHS Long‐Term Workforce Plan (NHS England 2023) set out the need to increase the number of nurses in the workforce by improving access to nursing careers.

The reported advantages of pre‐nursing VET Schemes concern progression into future nursing pathways. Recent empirical evidence from international contexts suggests that participation in pre‐nursing VET schemes fosters the motivation and desire to become a nurse through taking advantage of opportunities such as building relations with clinical staff (Rintala and Nokelainen 2020), interactions with patients, shifting perceptions on future career options, perceived self‐efficacy and having an evolving identity as a healthcare professional (Kyle et al. 2020; Smith et al. 2015). Previous VET schemes have been shown to benefit healthcare organisations, demonstrating a 60% progression rate into nursing (Norman et al. 2008). Pre‐nursing VET schemes can help learners transition to formal nurse education. They have also been found to widen access to people from Black, Asian, and ethnic minority backgrounds (Watson et al. 2005) in some instances.

The reported disadvantages of pre‐nursing VET schemes suggest scant evidence of their impact on widening participation, ongoing or future career trajectories, or learners' academic confidence.

Sometimes, they do not build academic confidence in those progressing to a nursing degree (Draper and Watson 2002). The Council of Deans of Health found limited evidence on how pre‐nursing VET schemes grow the healthcare workforce (Smith et al. 2015). Universal educational or practice standards for these schemes do not exist, and research has mainly focused on the learners' viewpoints. Generally, pre‐nursing VET studies are under‐researched and therefore reported. Thus, a deeper comprehension of the stakeholders' and learners' experiences and perspectives of pre‐nursing VET schemes on nursing workforce recruitment is required.

## 3 The Study

### 3.1 Aim(s) and Objective

The study aimed to evaluate learners' and stakeholders' experiences and perspectives on nursing workforce recruitment through two models of pre‐nursing VET schemes.

### 3.2 Secondary Objectives

To capture the experiences and expectations of learners undertaking two different pre‐nursing VET models on nursing workforce recruitment, secondary objectives were identified:

- To examine key stakeholders' experiences and expectations of the pre‐nursing VET schemes.
- To capture the experiences and expectations of learners undertaking both models and explore their views about the scheme, including recruitment, motivations, the extent of their learning in practice and opportunities/plans for their future employment/study.
- To interpret study findings through a social capital theory framework.

## 4 Method

### 4.1 Study Design and Setting

The study design was qualitative and consisted of using semi‐structured participant interviews. The study setting was a region covering four Southeast England National Health Service (NHS) trusts that implemented a pre‐nursing vocational cadet scheme in 2019. Participants in the scheme had three potential exit points: local employment as a health care assistant, access to nurse associate training or an undergraduate nursing degree programme. The scheme aimed to build a sense of belonging in the community, create more job opportunities, and attract people to join the NHS. Two models of the pre‐nursing VET scheme were implemented concurrently through a placement and employment model.

The placement model was aimed at students studying BTEC/T‐level qualifications in year 12/13 at school, alongside A′ levels or BTEC/T‐level qualifications alone through a local further education (FE) college. To meet the work‐based requirements of BTEC/T‐level study, placement model learners spent 1 day per week in a clinical placement organised by the school or FE college. The remaining 4 days were spent at their respective school or FE college, where they attended the academic component delivered by school or FE college‐employed tutors. However, school or FE tutors communicated with clinical placement staff to arrange student placements and provide ongoing support to learners on placement.

The employment model employed young people as ‘cadets’ in a senior healthcare support worker apprenticeship. Cadets were employed 4 days weekly in a specific clinical setting and received a salary. The academic component of the employment model was T‐Level qualifications underpinned by an apprenticeship standard. Learners in the employment model spent 4 days per week in their clinical setting. They received less classroom education (1 day per week, delivered by the employing healthcare trust) but more exposure to clinical placement than in the placement model.

No single organisation in the locality was responsible for the quality of the pre‐nursing VET schemes, with different local organisations leading their implementation, delivery and quality. However, staff within these organisations met regularly as a stakeholder group to share learning and consider the schemes' impact on recruitment to nursing and other healthcare careers.

### 4.2 Theoretical Framework

Social capital theory highlights the benefits of forming connections based on reciprocity, efficiency and trustworthiness (Putnam 2001). As a result, the framework is well‐suited for emphasising assets that foster employability and future career progression. Connections create intellectual and social assets for individuals and society, leading to higher well‐being and satisfaction in work‐based activities (Requena 2003). Social capital is collectively owned by the partners in a relationship, and no one has exclusive rights. As a theoretical lens, social capital can identify benefits and assets for society and individuals. To identify the enhancing resources, advantages, benefits or tensions in recruiting to and accessing nursing careers, it helps to consider stakeholders' and learners' experiences side by side.

A social capital theory framework considers three interrelated facets (Nahapiet and Ghoshal 1998): structural, relational and cognitive. The structural facet concerns the properties of a social system and how significant networks and ties become embedded. It offers a lens to examine stakeholder networks in securing and operationalising clinical placements for pre‐nursing VET learners. In turn, it can help understand how learners experience the created social structures, which may impact their learning and progression and become a source of ongoing feedback for stakeholders.

The relational facet refers to behavioural assets created and leveraged through relationships (Nahapiet and Ghoshal 1998). In the context of pre‐nursing VET schemes, it can help to evaluate how stakeholders build relations with learners to gain their trust and embed professional values and identities as future healthcare professionals. It can also further understand how stakeholders build relationships across organisational boundaries.

The cognitive facet focuses on the value of resources that can provide shared representativeness, interpretations and meanings between parties. These resources can also create assets for intellectual capital. In the context of pre‐nursing VET schemes, the cognitive facet can help evaluate how cognitive resources are built and embedded within social systems by stakeholders, how learners use them as a shared, professional language and how the community benefits from them. High levels of social capital in each of the three facets interconnected can lead to higher well‐being and satisfaction from work‐based activities (Requena 2003) for groups and individuals.

### 4.3 Sampling and Recruitment

Stakeholder recruitment to the study was through convenience sampling, assisted by the pre‐nursing VET scheme's regional project manager. An email flyer was sent to four healthcare trusts, and nine potential participants agreed to participate.

Seven stakeholder participants implemented the placement model, whereas two implemented the employment model. Each stakeholder had multiple responsibilities, including project management, placement organisation, monitoring, teaching, administration, pastoral care, recruitment and selection.

Through snowball sampling via the stakeholder participants, the potential of 22 trainee/learner participants who undertook a pre‐nursing VET scheme between 2020 and 2022 was possible. A sample of 8–12 participants was aimed for. Using snowball sampling, the study recruited eight pre‐nursing VET trainees from four regional schemes. The snowball method for sampling the trainee/learner participants was chosen because they were not a readily identifiable cohort. Four learners undertook the placement model, whereas the remaining four undertook the employment model. Stakeholders were unaware of which learners participated.

The stakeholder and pre‐nursing VET trainee participants represented each of the four NHS trusts in the region. The University research ethics committee granted ethical approval to conduct the study (study no HLS/2022/SS/14). Each participant provided written consent before participating in the study.

### 4.4 Data Collection

Data collection occurred between May and June 2022. Semi‐structured interviews were undertaken with stakeholders (pre‐nursing VET scheme administrators, educational course providers, NHS placement leads and a regional project manager) and pre‐nursing VET trainees undertaking the schemes. The research interviews were conducted online. Although COVID‐19 restrictions were officially lifted by then, many NHS organisations were restricted to only necessary face‐to‐face contact. All participants provided written consent beforehand. The interviews, lasting 30–60 min, were conducted by a study researcher (MW, RC or SN) through a topic guide (Appendix A) aligning with the study's goals. The questions for stakeholders aimed to capture their experiences and perspectives of two models of pre‐nursing VET schemes on nursing workforce recruitment by understanding and describing how the programmes were designed and delivered across the region within the context of the wider NHS system pathways. Interview questions for trainee/learner participants across both models aimed to capture their expectations and experiences of the programme, including its influences on their future aspirations and pathways.

### 4.5 Data Analysis

The study team transcribed all interview recordings verbatim for accuracy and returned them to the participants for checking. Two study researchers (MW and KG) thematically analysed the interview data. Firstly, an independent and complete interview transcript reading for familiarisation occurred. Secondly, a coding frame (Geisler 2018) was developed using the study research objectives as a priori codes within Microsoft Excel. The two researchers independently analysed the interview data using the coding frame to identify recurring codes and key messages (Boeije 2010). They interpreted the findings through the social capital theory framework lens, reaching a consensus about the final categories for inclusion (Table 1).

**TABLE 1:** Categories and codes interpreted through social capital theory.

Categories	Codes	Social capital theory facets
Expectations and motivation	*Possibilities for transition to different healthcare roles*	Structural
	*Workforce and clinical skill development*	Structural
	*Seeking appropriate post‐16 vocational education*	Structural
The relational aspects of curriculum delivery	*Negotiating placement opportunities*	Relational
	*Relational activities between stakeholders and cadets*	Relational
	*Stepped approach to teaching communication skills*	Relational
	*Developing a patient‐centred approach while seeking support and emerging self‐care*	Relational
Future possibilities	*Opportunities to work alongside others in the real world of work*	Cognitive
	*Possibilities for ongoing trajectories*	Cognitive
	*Securing concrete opportunities*	Cognitive
	*Implementing academic standards*	Cognitive
	*Impact of Covid‐19*	Cognitive
	*Managing workloads*	Cognitive
	*Benefits realisation for healthcare organisations*	Cognitive
	*Preparation for life and a future healthcare role*	Cognitive

### 4.6 Rigour

The research team, a group of nurses, reflected upon their recruitment into nursing and how initial access experiences were facilitated or inhibited by others through the progression from secondary education to undergraduate nursing. The team discussed any meaningful experiences and assumptions throughout the research planning, data collection and analyses to expose any underlying assumptions. Peer review, member checking and data triangulation were employed to ensure credibility (Lincoln and Guba 1985). The consolidated criteria for reporting qualitative research (COREQ) guidelines (Tong et al. 2007) were followed.

## 5 Findings

The key themes from the analysed interview data were ‘expectations and motivation’, ‘the relational aspects of curriculum design and delivery’ and ‘future possibilities’. As reported below, each theme represents both the stakeholders' and learners' perspectives.

### 5.1 Expectations and Motivation

In discussing their experiences in designing and implementing pre‐nursing VET schemes, stakeholder participants from both models described their motivations for creating a framework for aspirational learning. This included possibilities for future career transitions beyond nursing:

It's not about becoming a nurse but giving an insight into the NHS. We emphasise confidence and growth, insight into the NHS and career options instilled through the placement experience and for wherever they go in the future. (Placement model)

Stakeholder participants reported considering potential future healthcare job opportunities for learners. The placement model was stated to focus on creating future healthcare career options and access to higher education. However, the employment was reported to be aimed at recruiting and retaining a local workforce while helping learners catch up on secondary academic education. Both models aimed to develop learners' clinical skills and professional values, encourage resilience, and boost confidence. Stakeholder participants appeared equally motivated to support workforce development through both models and to give learners a better understanding of the NHS.

We clarify a job first and a course second, providing work and clinical skills. We start talking about career progression from the beginning. (Employment model)

The pre‐nursing VET trainee participants described how they sought alternative post‐16 compulsory education pathways to formal school education and how the pre‐nursing VET schemes appeared to offer these opportunities. Many learners reported joining a pre‐nursing VET programme for blended academic and work‐based learning. They anticipated learning about nursing specialities and working with experienced NHS nurses while improving their social skills. The programme was reported to meet learners' expectations and aspirations by connecting with different learning preferences.

I like to learn visually and practically. It seemed accessible. You could talk with senior nurses about possibilities and build confidence with socialising to grow as a person. (Placement model)

### 5.2 Relational Aspects of Curriculum Design and Delivery

Stakeholder participants reported that creating relationships was crucial to implementing the pre‐nursing curriculum. Stakeholders stressed the need for collaboration between project leaders, college administrators, tutors, placement leads and clinical staff to benefit all parties, including the pre‐nursing VET trainees. Stakeholders and administrators worked together to create placement opportunities, facing challenges like finding enough placements and maintaining/expanding opportunities. Pre‐nursing VET course tutors evaluated each learner's social and emotional maturity to determine placement timing while ensuring clinical staff could effectively support learning and development.

Managing an 18/19‐year‐old at university is distinct from the maturity and level of understanding of a 17‐year‐old cadet. We moved the placements to Year 2, so they go with more experience. (Placement model).

One initiative that was identified to promote learners' confidence involved creating placement buddies but this was reported to give cadet course leads and placement leads an additional responsibility for preparing placement buddies for their roles.

Stakeholder participants reported their interactions with prospective trainees through recruitment, selection and induction. They emphasised how trusting relationships were built through pastoral roles and classroom activities. Teaching approaches were described that considered trainees' age, clinical situations, professional values, communication, and patient care needs. Safe space discussion opportunities were described as helping to identify and assist struggling students.

The structure of a working environment is a culture shock, for example, drilling skills such as communicating with clinical areas by telephone. Debriefing offers the cadets an opportunity to talk. (Placement model)

Stakeholder participants noted that learner engagement with the curriculum's relational aspects led to increased confidence. However, stakeholders acknowledged the need to approach teaching the relevant skills incrementally. Described benefits for learners of this approach included developing coping techniques, finding their position within a healthcare team, and improving communication skills with patients while enhancing empathy and emotional reasoning:

Trainees placed in the paediatric ward got fantastic feedback. Some received the Chief Nurse Award, which is phenomenal. A patient's parent triggered one award. (Placement model).

Pre‐nursing VET trainee participants stated that they valued the relational aspects of patient‐centred care, such as developing social skills, self‐awareness and seeking emotional support. Both placement and employment models were reported as positive experiences where patient‐centred care involved physical care, communication skills, psychological support and handling challenging behaviour.

We've learnt about caring for patients, 6C's, compassion, empathy, the skills you need from learning a bedside manner to dealing with abuse, which is something we came across. (Placement model)

The pre‐nursing VET trainee participants identified that classroom learning was equally as important as the support from placement staff in reaching an understanding of patient‐centred care:

Learning in the lessons benefits the work because we can understand the patients more. (Employment model)

Pre‐nursing VET trainees also reported developing confidence in social skills as essential to evaluating their experiences, describing growing confidence to communicate with people across the lifespan and ask questions:

I learnt skills for my future. I am confident in what I do now. Many staff members supported me. When I looked at myself before, I didn't communicate well. I wasn't someone who'd be open and talk to people. (Employment model)

The pre‐nursing VET trainees described their ensuing strategies for seeking emotional and psychological support from tutors, placement leads, and clinical staff. They identified the learning activities that helped them to develop self‐care strategies, such as reflective practice and catch up sessions with tutors and placement leads:

My favourite thing about the scheme is the support. The nurses on the wards welcomed us. I had some horrible days. You see stuff in the hospital you haven't seen before. They'll talk you through it. (Placement model)

### 5.3 Future Possibilities

Future possibilities for all involved in the cadet schemes emerged through stakeholders' and pre‐nursing VET trainees' reported emergent knowledge. They described what worked well, what needed improving and the potential benefits of the schemes for stakeholder organisations and their serving communities. Stakeholder participants reported that offering learners placement opportunities where they worked alongside healthcare professionals broadened learners' horizons, providing them with insights into future healthcare career options. They emphasised the benefits of real‐world work experience, including collaboration in intense environments and an understanding of the NHS.

Exposure provides authentic pathways into healthcare roles. Some trainees want to be army nurses, paramedics, children's nurses or occupational therapists. They develop ideas about roles once they have first‐hand experience. (Placement model)

One stakeholder commented that they had evaluated the 22 graduated trainees' career paths to reflect on how their work experiences impacted future trajectories. Of these, one had pursued physiotherapy, eight had pursued associate nursing apprenticeships, and the remainder had pursued nursing degrees through placement or direct employment.

Stakeholder participants described challenges in implementing plans relating to the schemes because of a lack of placements. Departments needed more staff before accommodating trainees, and competition from other programmes made it more complex. To expand options, some suggested exploring placement opportunities beyond physical healthcare services. However, participants noted a caveat around the legal and professional restrictions concerning minors, restricting the hours spent in practice and in accessing areas such as mental health.

I would love the dedicated time to explore other services. Some [areas] are unsuitable for young people to enter as trainees. Equally, some would be [okay]. (Placement model)

Course leads reported struggled with implementing academic standards and assessing trainees because of unclear guidelines. Some stakeholder participants (employment model) questioned the logic of educational standards, resulting in the over‐assessment of cadets because of apprenticeship and BTEC requirements.

It is convoluted regarding the trainees' assessment and the cross‐over between apprenticeship standards and the diploma (Employment model).

While some trainees reported struggling to balance academic and placement workloads, stakeholder participants noted that communication between course leaders and placement areas often helped address non‐attendance issues among trainees. Dropping out of placement attendance was reported as problematic for some trainees, primarily in the placement model.

If they're tired, the placement goes first. They think they can get away with it. It's extending and deepening the communication between the hospital and us, all easily achievable. (Placement model)

Pre‐nursing VET trainee participants shared how they balanced academics and practical aspects. They attended well‐being sessions, sought peer help and faced challenges when limited placement opportunities affected learning.

I've been in two different placements because I had to move to sign competencies off. (Employment model)

Stakeholder participants from both models expressed their intentions to expand access to the VET schemes to those not usually pursuing healthcare career opportunities. This included individuals from local communities and those with disabilities. However, they acknowledged the need for more inclusivity, as most cadets were female and White.

We can grow our own, and that's fantastic. We should widen the potential of our young people who don't get the chance and be inclusive (Placement model).

Participants reported the benefits of the schemes for local communities, including recruiting individuals to the local healthcare workforce. Stakeholder participants identified this as a key goal of the schemes.

The benefits are long‐term. We're providing a bulk of young people inducted into the organisation, we're filling jobs at the end of the day, providing care. (Placement model)

In the placement model, stakeholder participants described pre‐nursing VET schemes as crucial for providing further education, health and social care career opportunities. Trainees were perceived as valuable resources in clinical placements, learning from placement staff while contributing to staff development. The schemes were reported as aligning with the ethos of educational institutions, helping students develop as a community.

Just having a young person's eyes. We encourage their feedback to placement buddies. Some things changed as a result. The buddies grow in confidence and self‐esteem. We know that brings good patient interactions and better outcomes. (Placement model)

Adapting schemes to learners' needs was identified as a way of offering stakeholders professional development opportunities, such as undertaking a postgraduate education certificate. Stakeholders said that they also received positive feedback about the cadets from placement areas, including benefits beyond clinical performance.

Trainees bring youthful enthusiasm and energy to the wards, which is warmly received. They work hard, which benefits patients and colleagues. (Employment model)

Trainee participants from both programme models identified the positive influences of the schemes on their learning and career paths. Classroom instruction provided confidence to understand clinical context and terminology, smoothing the transition into placement.

It provides a framework for placement. One of our units was dealing with children with additional needs. I used that when I went onto the ward and helped a child deal with barriers. I gave him sensory toys. Learning in the classroom built my knowledge to care for this child. (Placement model)

Pre‐nursing VET trainee participants stated how they had reflected on their personal growth over the programme and found it valuable preparation for the future. They reported gaining practical experience in critical situations, such as working in mental health and learning to react skilfully. Mistakes, honesty, and seeking help were identified by trainees as crucial to personal development.

Be honest when you are struggling…. it is a new experience Remember that you're looking after the patients as much as yourself. (Employment model)

The trainee participants identified critical influences on their future trajectories. These included opportunities to plan career pathways, apply learnt skills to university and nurse associate applications, and enhance their academic qualifications. Bespoke placements during the pre‐nursing VET scheme were reported to influence trainees' potential career choices and provide useful experience, which they could evidence on their curriculum vitae.

## 6 Discussion

This study aimed to evaluate learners' and stakeholders' experiences and perspectives of two models of pre‐nursing VET schemes on nursing workforce recruitment.

Our findings have been interpreted through a social capital theory lens.

The first key finding showed the expectations and motivations of stakeholders and learners and how they became aligned through the stakeholder's motivation to create a framework for aspirational learning and the learner's expectations for alternative post‐16 compulsory education pathways.

The study's findings showed the stakeholders' (project managers, college, school tutors and placement leads) passion for early nursing career exposure. Stakeholder participants reported aspiring to create informed choices through a pre‐nursing VET scheme. Commonalities in each of the pre‐nursing VET scheme models showed effective partnerships between education and healthcare organisations to create pre‐nursing contexts essential to attracting young people to careers in nursing (Thompson et al. 2001).

The learner participants often sought alternative post‐16 compulsory education pathways to formal school education. Their expectations for real‐world healthcare work experience were met when critical practice‐based relationships with clinical staff and patients developed through employing emotional intelligence and empathetic reasoning. These emergent resources can help individuals to grow within a community of practice (Lave and Wenger 1991), form meaningful relationships and develop vocational skills and professional identities. Peer‐group meetings have also been shown to be effective in helping learners by creating exemplary vocational learning environments (Bouw et al. 2019).

The second key finding showed how the relational aspects of curriculum design and delivery benefitted the learners' emotional, social and cognitive development. The stakeholders' efforts to develop the structural facets of the respective cadet schemes were clearly identified in the findings and cannot be underestimated. Examples included efforts to coordinate academic and clinical environments by linking college/school course tutors with placement leads and identifying different career options through placement experiences. Billett (2006) recommended that workplace curricula, workforce and clinical skill development were best established through strong social structures between academic and clinical environments. These links can enable stakeholders to tailor their requirements and identify which local resources and policies need to be developed to optimise course structures.

Despite their recent implementation, the pre‐nursing VET schemes reported on showed how stakeholders can configure a network of interaction and interdependence to build social capital (Nahapiet and Ghoshal 1998). The needs of diverse post‐16 learners were considered by creating appropriate academic and placement learning environments to develop skills and confidence for future healthcare career options, trajectories and work. In creating social capital, stakeholders can become attuned to the developmental transitions made by cadets during their time on the scheme. Stakeholders' perceptions of the learners' transitions aligned with the learners' descriptions of self‐reflection on their emotional, social and cognitive progression by leveraging assets through the schemes.

The third key finding suggested that all involved in the cadet schemes developed emergent knowledge about the future possibilities for both the learners and the ongoing schemes. The findings emerged from the participants' reflections on their experiences and perceptions about what worked well for everyone, the benefits for stakeholder organisations and their serving communities, and what could be improved. The findings highlighted that the resources required to make choices can create social capital for learners. Over a third of the first cohort across the schemes opted for a nursing associate pathway following the pre‐nursing VET scheme. This option gave students more time to decide if progressing to a nursing career was an appropriate choice while concurrently offering financial stability and the potential to progress to a shortened nursing degree. This flexibility is critical in unfavourable economic times, encouraging young adults to stay in the workforce while they consider the most appropriate career pathway for them. The study participants were working and living in one of the most expensive regions in the UK, adding to pressures relating to the recruitment and retention of staff. In addition, the withdrawal of the student bursary for UK‐based pre‐registration nurses in 2016 has led to a decline. It is considered a factor that has led to a steady decrease in nursing applications of up to 18% between 2022 and 2023 (White 2023). Having the option to undertake a shorter nursing degree programme might be attractive to students with financial pressures. It may be a deciding factor in their decisions about whether to enrol.

Learners in this study reported actively participating in and learning through the social capital afforded to them, shaping their motivations and values for a career‐focused education. Some trainees made nuanced career choices, such as pursuing physiotherapy, midwifery or children's nursing, which is likely to be influenced by the strong partnership between the academic and placement settings they were working within.

Areas that required improvement were reported concerning placement opportunities, clarity about the academic standards underpinning the programme and widening participation.

Both stakeholders and learners described difficulties in accessing clinical placement opportunities. In the employment model, learners were employed in a specific trust healthcare assistant role, which made up the placement experience. Although trust‐based tutors endeavoured to provide structured activities to overcome obstacles, some clinical areas were specialised, restricting the clinical skills that could be learned and practised. Some placement areas, for example, mental health, were not permitted for learners under 18 years. The placement model allowed for increased flexibility, even with reduced time dedicated to clinical placement, relying on school or college tutors to identify potential placement opportunities. The trainee's evaluation of the schemes highlighted a deep motivation to pursue a vocationally oriented education to shape future career choices. Our findings support other studies highlighting the importance of placement experiences in developing cadets' skills and confidence, while changing perceptions of potential future nursing roles (Beattie et al. 2014; Kyle et al. 2020; Smith et al. 2015). However, few studies have followed VET trainees as they progress into pre‐registration nursing schemes, and those that have highlighted the criticality of the practical preparation gained through prior placement experience (Draper and Watson 2002; Norman et al. 2008; Slator 2010). Our findings reinforce the idea that placement experiences are crucial for cadets' skill development, confidence, and perception of future nursing roles (Beattie et al. 2014; Smith et al. 2015).

A pre‐nursing VET scheme is only one entry point for future potential nursing careers. Although the vocational qualifications obtained may enable entry to a future nursing course, their equivalence to formal secondary school qualifications is unclear. The recently implemented UK T‐levels are reported as unsatisfactory due to their content and structure, as well as challenges in content delivery (OFSTED 2023). In our study, the stakeholders delivering the employment model felt that the students were over‐assessed because it was necessary to achieve learning outcomes associated with BTEC and an apprenticeship standard with no direct learning gain for the pre‐nursing VET trainees in doing both. Furthermore, the study requirements were burdensome, given that trainees were employed 4 days per week. Some evidence suggests that pre‐nursing VET schemes do not instil academic confidence in those who go on to higher education to pursue a nursing degree (Draper and Watson 2002; Slator 2010).

The demographic profile of the majority of VET scheme trainees is female and White. The NHS long‐term Workforce Plan (NHS England 2023) proposes a 92% increase in adult nursing training places by 2032. Thus, pre‐nursing VET schemes have a pivotal role in creating social capital for candidates who would not traditionally choose a career in nursing or healthcare. Although pre‐nursing VET schemes are aimed at young people, the number of students over the age of 21 years accessing pre‐registration nursing over the last decade has declined (White 2023). This decline may infer that more mature students are choosing not to consider nursing degree programmes as viable career choices. Expanding the age range of VET schemes to consider older learners may be one way to encourage more mature students into the profession.

### 6.1 Strengths and Limitations of the Study

This study contributes by showing how pre‐nursing VET schemes can potentially improve career planning, higher education and job opportunities. Uniquely, it showed the experiences and perspectives of stakeholders and their motivations and expectations to design and deliver a curriculum that aligned with learners' motivations and expectations, leading to social capital for learners. Such social capital benefits society and instils a sense of achievement in young people during a formative period. According to Zittoun (2003), pursuing a vocational path early on can help individuals who experience academic setbacks, and successful transitions can provide emotional and psychological healing. Crafter et al. (2019) highlighted the transition from education to work as a significant milestone involving important decisions with potential long‐term consequences. These transitions are unique to each individual. However, young people need the skills to navigate career pathways. Our findings describe how pre‐nursing VET trainees leveraged experiences to pursue future careers in healthcare, and that the skills learned may also benefit those who pursue alternative career paths. Although the UK context differs from other countries and regions, the recruitment crisis in nursing is an international concern including project management, placement organisation, monitoring, teaching, administration, pastoral care, recruitment and selection. The social capital lens can be transferred to other pre‐nursing VET contexts.

The pre‐nursing VET participants described how they sought alternative post‐16 compulsory education pathways to formal school education and voiced their aspirations for a vocational health career. The study provides insight into the lack of recognition of this route to nursing. This study reported the experiences and perspectives of stakeholders and trainees within two different pre‐nursing VET models, which highlighted the lack of standardisation in access and educational delivery, which has implications for learners. There were limitations to widening participation, placement opportunities and understanding how the schemes prepare learners academically.

A limitation of the study is that we did not collect any sociodemographic data from participants. The study could be strengthened through additional research methods correlating the relationships between variables such as social and economic factors, satisfaction with undertaking a pre‐nursing VET scheme, and ongoing career choices. Slator (2010) argues that pre‐nursing trainees' family orientations towards careers influence individuals' choices about pursuing vocational education pathways. Although some participants touched upon family members' roles in helping them choose a pre‐nursing VET scheme, we did not explore this in depth.

Our research in a UK region used convenience and snowball sampling, which may be biased towards those favouring nursing career interests. Gathering data from individuals who found the schemes challenging or unsatisfactory could have enhanced the study by offering more diverse perspectives.

### 6.2 Recommendations for Further Research, Policy, and Practice

Stakeholders play a crucial role in successfully implementing pre‐nursing VET schemes, which link with social and economic policy objectives (Culley and Genders 2003; Smith et al. 2015). Allowing sufficient time to embed and sustain such schemes is crucial. Given the limited evidence base, systematically evaluating and analysing the benefits for all parties is essential to understanding a scheme's success.

Our study focused on two models of pre‐nursing VET schemes provided by educational or healthcare organisations. The study highlighted the critical role of stakeholders in designing, implementing and delivering different models. Widening access to nursing careers is a current healthcare policy aim (NHS England 2023), which pre‐nursing VET schemes can help to address. The study highlighted the limitations of the reported schemes due to a lack of standardisation in access and academic delivery. In the UK, pre‐nursing VET schemes appear to have a resurgence. However, there does not appear to be any central policy direction or standardisation of such schemes, which are areas for attention.

The pre‐nursing VET schemes in the study were implemented in one region of the UK to address local healthcare workforce recruitment issues. Further studies are required to compare with other regions nationally and internationally to determine how pre‐nursing VET schemes significantly address the global nurse recruitment crisis while considering local social and economic contexts. This includes the extent to which socioeconomic factors affect learners' career choices.

A dearth of longitudinal evidence exists on how pre‐nursing VET schemes impact learners pursuing a nursing or other healthcare career through the various routes available. A recent Norwegian study (Drange and Ingelsrud 2023) focused on career choices over 10 years after vocational training in licensed practice nursing. The researchers highlighted the importance of early‐career employment opportunities for promoting nurse retention. Tracking pre‐nursing VET trainees pursuing healthcare careers after participating is essential to evaluate the effectiveness of pre‐nursing VET schemes in contributing to the long‐term recruitment and retention of nurses and healthcare professionals.

## 7 Conclusion

This study aimed to evaluate learners' and stakeholders' experiences and perspectives of two models of pre‐nursing VET schemes on nursing workforce recruitment. It showed an alignment between stakeholders' and learners' expectations and motivations, building trusted relationships across organisational boundaries, and developing shared cognitive resources to help shape future healthcare professionals. Cognitive resources, such as professional language and codes, benefit learners, stakeholders and the clinical healthcare community, potentially expanding access and recruitment to nursing and healthcare careers. Identifying effective strategies for creating such career opportunities is important for long‐term sustainability. However, the study also showed limitations of the pre‐nursing VET schemes that were evaluated including a lack of standardisation in access and the value of academic preparation for ongoing nurse education. Pre‐nursing VET schemes require policy, research and educational investment to evidence their potential value in creating opportunities for future employment, academic achievement, confidence and recruitment to nursing careers. Furthermore, a deeper understanding of the socioeconomic factors affecting learners' nursing and healthcare career choices can enhance existing knowledge.

## Author Contributions

**Marion Waite:** conceptualisation, analysis, interpretation, drafting the work and revising it for important intellectual content, final approval of the version to be published and accountability for accuracy and integrity. **Jane Appleton:** study design, conceptualisation, analysis, interpretation, revising the work for important intellectual content, final approval of the version to be published and accountability for accuracy and integrity. **Kathleen Greenway:** conceptualisation, analysis, interpretation, revising the work for important intellectual content, final approval of the version to be published and accountability for accuracy and integrity. **Robert Crookston:** acquisition of data and data analysis and interpretation. **Sanj Nathoo:** acquisition of data. **Catherine Henshall:** conceptualisation, analysis, interpretation, revising the work for important intellectual content, final approval of the version to be published and accountability for accuracy and integrity.

## Conflicts of Interest

The authors declare no conflicts of interest.

## Supporting information


Data S1.

## Data Availability

The data that support the findings of this study are available on request from the corresponding author. The data are not publicly available due to privacy or ethical restrictions.

## Appendix A One‐Topic Guide

Study title: Workforce recruitment through pre‐nursing vocational and education training schemes. A qualitative evaluation through a social capital lens.

Interview protocol stakeholder participants:

1. Can you describe the design and delivery of your programme?
2. Describe your role within the course delivery.
3. Can you describe the pathway the students take through the course?
4. What opportunities does the course offer a student?
5. What aspects of the course need improvement or change in the future and why?
6. Can you identify any constraints for running the scheme?
7. What opportunities does this course offer for widening participation in healthcare careers?
8. What are the benefits to the organisation/Trust and staff in delivering this course?
9. What are the adaptations or impact of Covid 19 on the course
10. Which healthcare career(s) do you feel this cadetship/course best aligns with and why?
11. Are you aware of the destinations of attendees of the course?
12. Is there anything else you would like to share with us about the course?

Interview protocol nurse cadet participants:

1. What first attracted you to the Nurse Cadet Scheme?
2. What made you apply for the Nurse Cadet scheme?
3. What made you decide to pursue this course over other courses?
4. How has the course prepared you for your experiences in practice?
5. How do you think what you learn in the classroom relates to what you see on placement?
6. How have you managed the workload of the course?
7. What (if anything) would you like to see in the course that is not there now?
8. Do you think the balance between theory and practice in the course is right?
9. What kind of support has been available to you in practice and in theory?
10. How has/will the course guide you towards your chosen career path?
11. What would you say to others who are considering taking the course?
12. If you could speak to yourself before starting the course, what advice would you give yourself?
