# Supplementary material for: Workforce Recruitment Through Pre‐Nursing Vocational and Education Training Schemes. A Qualitative Evaluation Through a Social Capital Lens
Source: Nurs Open. 2025 Feb 9;12(2):e70159. doi: 10.1002/nop2.70159 (PMC11807385; doi:10.1002/nop2.70159)
Supplement: Supplementary file 1 — Data S1. [file NOP2-12-e70159-s001.docx]

Table 1 Categories and codes interpreted through social capital theory.

| **Categories** | **Codes** | **Social capital theory facets** |
| --- | --- | --- |
| Expectations and motivation | *Possibilities for transition to different healthcare roles* | Structural |
|  | *Workforce and clinical skill development* | Structural |
|  | *Seeking out appropriate post-16 vocational education* | Structural |
| The relational aspects of curriculum delivery | *Negotiating placement opportunities* | Relational |
|  | *Relational activities between stakeholders and cadets* | Relational |
|  | *Stepped approach to teaching communication skills* | Relational |
|  | *Developing a patient-centred approach while seeking support and emerging self-care* | Relational |
| Future possibilities | *Opportunities to work alongside others in the real world of work* | Cognitive |
|  | *Possibilities for ongoing trajectories* | Cognitive |
|  | *Securing concrete opportunities* | Cognitive |
|  | *Implementing academic standards* | Cognitive |
|  | *Impact of Covid-19* | Cognitive |
|  | *Managing workloads* | Cognitive |
|  | *Benefits realisation for healthcare organisations* | Cognitive |
|  | *Preparation for life and a future healthcare role* | Cognitive |
